# Reproductive Outcomes of Women with Turner Syndrome Undergoing Oocyte Vitrification: A Retrospective Multicenter Cohort Study

**DOI:** 10.3390/jcm12206502

**Published:** 2023-10-13

**Authors:** Sapthami Nadesapillai, Femke Mol, Simone L. Broer, Linda B. P. M. Stevens Brentjens, Marieke O. Verhoeven, Karst Y. Heida, Mariëtte Goddijn, Ron J. T. van Golde, Annelies M. E. Bos, Sanne van der Coelen, Ronald Peek, Didi D. M. Braat, Janielle A. E. M. van der Velden, Kathrin Fleischer

**Affiliations:** 1 Department of Obstetrics and Gynecology, Radboud University Medical Center, 6500 HB Nijmegen, The Netherlands; 2 Department of Obstetrics and Gynecology, Amsterdam University Medical Center, Center for Reproductive Medicine, Amsterdam Reproduction & Development Research Institute, University of Amsterdam, 1100 DD Amsterdam, The Netherlands; 3 Department of Reproductive Medicine, University Medical Center Utrecht, 3508 GA Utrecht, The Netherlands; 4 Department of Obstetrics and Gynecology, Maastricht University Medical Center+, 6229 HX Maastricht, The Netherlands; 5 GROW School for Oncology and Reproduction, 6229 ER Maastricht, The Netherlands; 6 Dijklander Hospital, Centrum Voor Kinderwens, 1441 RN Purmerend, The Netherlands; 7 Department of Pediatrics, Amalia’s Children’s Hospital, Radboud University Medical Center, 6500 HB Nijmegen, The Netherlands; 8 Department of Reproductive Medicine, Nij Geertgen Center for Fertility, 5424 SM Elsendorp, The Netherlands

**Keywords:** Turner syndrome, oocyte vitrification, fertility preservation, pregnancy

## Abstract

Background: Turner syndrome (TS) is accompanied with premature ovarian insufficiency. Oocyte vitrification is an established method to preserve fertility. However, data on the oocyte yield in women with TS who vitrify their oocytes and the return rate to utilize the oocytes are scarce. Methods: Retrospective multicenter cohort study. Data was collected from medical records of women with TS who started oocyte vitrification between 2010 and 2021. Results: Thirty-three women were included. The median cumulative number of vitrified oocytes was 20 per woman. Complications occurred in 4% of the cycles. Significant correlations were found between the cumulative number of vitrified oocytes and AMH (r = 0.54 and *p* < 0.01), AFC (r = 0.49 and *p* < 0.01), percentage of 46,XX cells (r = 0.49 and *p* < 0.01), and FSH (r = −0.65 and *p* < 0.01). Spontaneous (*n* = 8) and IVF (*n* = 2) pregnancies occurred in 10 women ± three years after vitrification. So far, none of the women have returned to utilize their vitrified oocytes. Conclusions: Oocyte vitrification is a feasible fertility preservation option for women with TS, particularly in those with 46,XX cell lines or sufficient ovarian reserve. Multiple stimulation cycles are recommended to reach an adequate number of vitrified oocytes for pregnancy. It is too early to draw conclusions about the utilization of vitrified oocytes in women with TS.

## 1. Introduction

Turner syndrome (TS) is a genetic condition affecting one in 2500 girls [1]. The partial or complete loss of one sex chromosome causes premature ovarian insufficiency in the majority of girls with TS [2]. The accelerated loss of follicles in women with TS starts from 14 to 18 weeks of gestation and continues until the ovarian reserve is fully depleted, usually during childhood or early adolescence [3,4]. The pathophysiology of germ cell apoptosis and impaired folliculogenesis in girls with TS and the timeline at which this occurs is less clear and may be different for each woman with TS [2,3]. Women with a mosaic 45,X/46,XX karyotype are likely to have a higher ovarian reserve compared to women with a 45,X karyotype [3]. In general, the chance of having biological children is limited in women with TS and occurs in 2–13% [5,6]. Therefore, most women with TS rely on alternative options, such as oocyte donation or adoption, to become a parent in the future [7].

Most women with TS consider infertility the heaviest burden they have to bear during their lifespan [8]. Currently, ovarian tissue cryopreservation (OTC) and vitrification of mature oocytes are performed in girls and women with TS to preserve their fertility as early as possible. OTC can be performed at a young age without requiring ovarian activity, which could be beneficial for prepubertal girls with TS [7]. However, it is still considered an experimental procedure in girls with TS since there is no evidence that OTC will enhance the likelihood of pregnancies in this group [9,10].

Vitrification of mature oocytes is, therefore, the most applied treatment for women with TS to preserve their fertility, but also has limitations [9,11]. Firstly, the majority of women with TS have a low ovarian reserve, and, therefore, a lower oocyte yield at oocyte retrieval is to be expected [12,13]. Secondly, the higher risk of miscarriages in women with TS necessitates more vitrified oocytes to be stored for an ongoing pregnancy compared to women in the general population [5]. Finally, girls with TS should be emotionally and physically mature enough to undergo this treatment due to the stress and burden that this treatment may entail [7].

Publications on vitrification of oocytes and subsequent reproductive outcomes in young girls and women with TS are currently scarce. In past years, approximately 50 cases have been reported concerning the outcomes of oocyte vitrification in girls and women with TS [13,14,15]. So far, only one case has been published, describing a woman with 45,X/46,XX TS who became pregnant by using her own vitrified oocytes [16].

As data on oocyte vitrification in women with TS are still limited, we conducted a retrospective multicenter cohort study with the aim of gaining more insights into the reproductive outcomes of women with TS undergoing oocyte vitrification. In addition, we evaluated whether women with TS used their vitrified oocytes to become pregnant.

## 2. Materials and Methods

### 2.1. Study Design and Participants

A retrospective cohort study was conducted based on data obtained from five university hospitals in the Netherlands. Women with monosomic TS (45,X), mosaic TS (45,X/46,XX or 45,X/47,XXX), or structural aberrations of the X chromosome (e.g., isochromosome, X-deletion, or ring chromosome) who started an oocyte vitrification cycle between 2010 and 2021 were included. This study was not considered subject to the Dutch Medical Research with Human Subjects Law (WMO), as stated, and, therefore, no further medical approval was required by the local Ethical Committee of Nijmegen (2021-7322, 19 April 2021). Written informed consent was obtained if patients had not previously indicated that data in their medical record could anonymously be used for research purposes or if additional information regarding pregnancy outcomes was requested by questionnaire.

### 2.2. Data Collection

Data regarding patient characteristics (e.g., age, karyotype, menstruation cycle), antral follicle count (AFC), serum levels of follicle stimulating hormone (FSH), anti-Mullerian hormone (AMH) and estradiol prior to oocyte vitrification, and ovarian stimulation outcomes were collected from medical records by the local researchers from the different medical centers. Pregnancy outcomes were also retrieved from medical records, if known. In case pregnancy outcomes were not available, a questionnaire was sent by the local researcher to obtain data from the patients. Castor EDC EU HQ database (Amsterdam, the Netherlands, version 1.4.1) was used to combine pseudonymized data from all medical centers.

### 2.3. Outcome Measure

Primary outcome measures included the number of cryopreserved mature oocytes (metaphase 2) per cycle, number of pregnancies per cycle, and number of spontaneous pregnancies. Secondary outcome measures included patient’s age during stimulation, karyotype, hormone levels prior to stimulation, type of vitrification protocol, total FSH dose used for stimulation, number of oocytes obtained from oocyte retrieval, complications during stimulation/oocyte retrieval, interval between ovarian stimulation and usage of vitrified oocytes, and pregnancy outcomes (time to pregnancy, miscarriage, comorbidity during pregnancy, delivery, and postpartum and child’s health).

### 2.4. Statistical Analysis

Statistical analysis was performed using SPSS for Windows, version 27 (IBM Corp, Armonk, NY, United States). Baseline characteristics and continuous variables were expressed as mean ± standard deviation (SD) or medians with range/interquartile range (IQR) in case of non-normal distribution (Shapiro–Wilk). Categorical outcomes were reported as percentages.

The correlation between the number of vitrified oocytes and continuous variables was determined using Pearson’s correlation (r). Continuous variables were log transformed in case of a non-normal distribution. A *p*-value of ≤0.05 was considered statistically significant. 

## 3. Results

### 3.1. Baseline Characteristics

The patient characteristics of 33 women with TS who had had fertility preservation counselling and at least one ovarian stimulation cycle for oocyte vitrification are summarized in Table 1. The mean age at counselling was 20 ± 4.3 years. Karyotyping was performed on lymphocytes in 32/33 women (97%) and on buccal cells in 11/33 women (33%). The percentage of 46,XX cells in lymphocytes was primarily used for analysis, unless the 46,XX cell line was only present in buccal cells. In total, 20/33 women (61%) had a 46,XX cell line in lymphocytes or buccal cells, 9/33 women (27%) had structural aberrations (X-deletion (*n* = 3), isochromosome (*n* = 2), and ring chromosome (*n* = 4)), and 4/33 women (12%) had a 45,X/47,XXX karyotype. No women with 45,X monosomy were reported. In 8/33 women (24%), cardiac comorbidities were reported (e.g., aorta dilatation, bicuspid aortic valve, and mild aorta insufficiency). None of these women had a contra-indication for pregnancy. Other comorbidities were reported in 9/33 women (27%), including asthma, ulcerative colitis, kidney problems, depression, and thyroid disease.

Spontaneous menarche occurred in 32/33 women (97%). Ultrasound showed normal ovaries in 28/33 women (85%). Two women (#21 and #23) had ovarian cysts, one woman (#6) only had one ovary (after unilateral ovariectomy for OTC), one woman had a polycystic ovary (#11), and one woman had ovaries without signs of activity (#10). The median AFC was: 13 (range 1–48) in women with a 46,XX cell line (*n* = 19), 11 (range 0–26) in women with structural aberrations (*n* = 9), and 3 in two women with 45,X/47,XXX. Median serum levels of FSH, AMH, and estradiol per karyotype are shown in Table 2.

### 3.2. Outcome of Ovarian Stimulation and Vitrification of Oocytes

**Figure 1:**
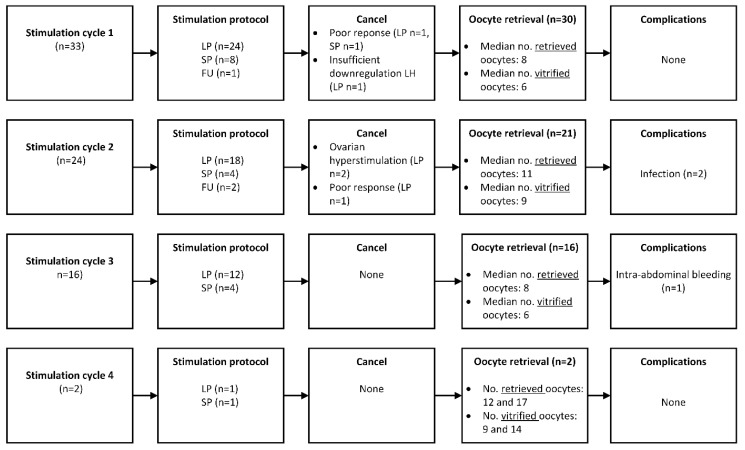
Overview of ovarian stimulation cycles and oocyte retrieval in 33 women with TS. LP = long protocol, using GnRH agonist with recombinant FSH or human menopausal gonadotropin or urofollitropin. SP = short protocol, using GnRH antagonist with recombinant FSH or human menopausal gonadotropin or urofollitropin. FU = flare up protocol, using GnRH agonist with recombinant FSH or human menopausal gonadotropin or urofollitropin. LH = luteinizing hormone.

An overview of all ovarian stimulation cycles is presented in Figure 1. The mean age was 21 ± 4.3 years at the start of the first stimulation cycle. The median daily dose of FSH/FSH + LH was 225 IE (range 50–450 IE). Human chorionic gonadotropin or GnRH agonist was used to start the ovulation process. Stimulation was canceled in two women due to poor response (#5 and #33) or insufficient downregulation of LH during the stimulation (#26).

Oocyte retrieval was performed in 30/33 women (91%). No oocytes were found after oocyte retrieval in one woman (#10). In the first stimulation cycle, the median number of retrieved oocytes was eight (range 0–29 oocytes) and the median number of vitrified oocytes was six (range 0–27 oocytes). No complications occurred during the first ovarian stimulation cycle.

**Figure 2:**
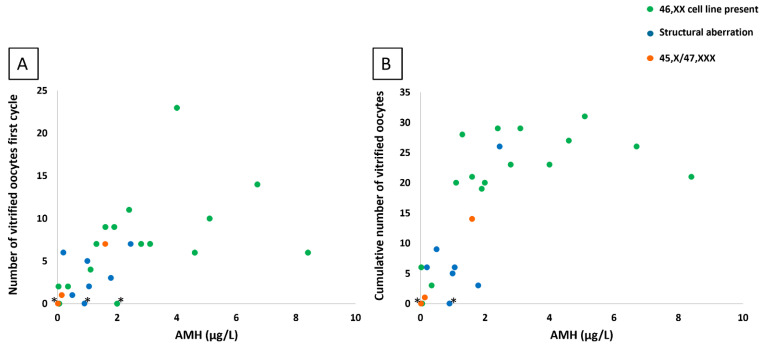
AMH levels and number of vitrified oocytes. Baseline AMH levels and the number of vitrified oocytes after the first stimulation cycle (**A**) and cumulative number of vitrified oocytes (**B**) of 26 women with TS are presented. In most women, AMH < 1 µg/L corresponded with ≤5 vitrified oocytes in the first cycle and <10 vitrified oocytes in total. The number of vitrified oocytes varied widely in women with an AMH ≥1 µg/L. Green dots represent women with a 46,XX cell line, blue dots represent women with structural aberrations, and orange dots represent women with 45,X/47,XXX karyotype. Stimulations that were cancelled are indicated with *.

A significant positive correlation was found between the number of vitrified oocytes in the first cycle and AMH (r = 0.51 and *p* = 0.01) (Figure 2A), AFC (r = 0.52 and *p* < 0.01), and percentage 46,XX cells (r = 0.38 and *p* = 0.04). No significant correlation was found between the number of vitrified oocytes in the first cycle, FSH level (r = 0.19 and *p* = 0.44), estradiol level (r = −0.24 and *p* = 0.39), or age (r = 0.23 and *p* = 0.21).

In total, 24/33 women (73%) underwent a second stimulation cycle. The median dose of FSH/FSH + LH was 225 IE (range 75–450 IE). Stimulation was canceled in two women due to ovarian hyperstimulation (#1 and #13) and in one woman due to poor response (#26). Twenty-one women had an oocyte retrieval (88%). The median number of retrieved oocytes was 11 (range 1–20 oocytes) and nine for vitrified oocytes (range 1–18 oocytes). Two women developed an infection after oocyte retrieval, one of whom was hospitalized because of a pyelonephritis. They were treated with antibiotics and fully recovered.

A third stimulation cycle was performed in 16/24 women (67%). The median dose of FSH/FSH + LH was 225 IE (range 50–450 IE). The median number of retrieved oocytes was eight (range 3–23 oocytes), and six for vitrified oocytes (range 3–15 oocytes). One woman had an intra-abdominal bleeding after oocyte retrieval which required a laparoscopic intervention; she fully recovered.

Only two women (#1 and #8) had a fourth stimulation cycle (2/16; 13%). One woman had a short protocol where 12 oocytes were retrieved, of which nine oocytes were vitrified. The other woman had a long protocol where 17 oocytes were retrieved and 14 oocytes were vitrified. No complications occurred.

**Figure 3:**
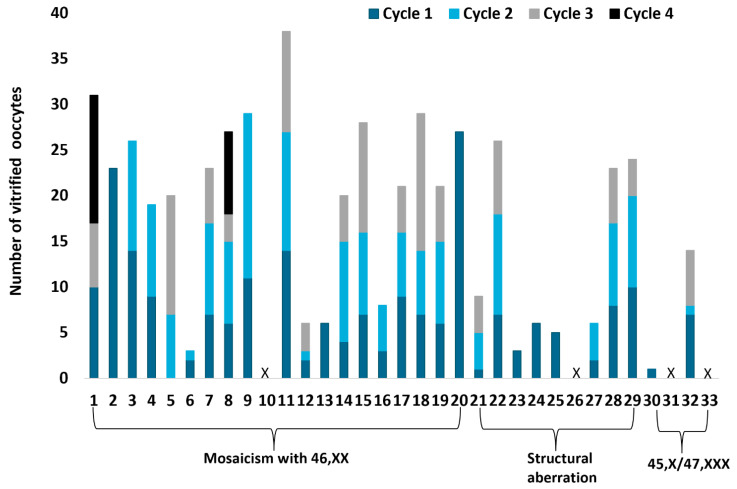
Number of vitrified oocytes per stimulation cycle and karyotype. An overview of 75 stimulation cycles of 33 women with TS is presented. Dark blue bars represent the number of vitrified oocytes after the first cycle, light blue bars after the second cycle, grey bar after the third cycle, and black bars after the fourth cycle. X indicates that no oocytes were vitrified.

In summary, 75 stimulation cycles were initiated, of which six did not result in an oocyte retrieval (8%) due to poor response, ovarian hyperstimulation, or insufficient downregulation of LH. Oocyte retrieval was performed in 69 cycles (Figure 3). Complications occurred in three cycles (4%). The median cumulative number of vitrified oocytes was 20 (range 0–38 vitrified oocytes). In total, 17/33 (52%) women vitrified ≥20 mature oocytes. 

**Figure 4:**
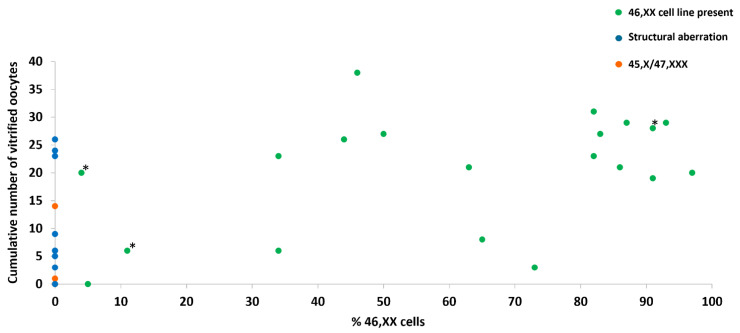
Cumulative number of vitrified oocytes and percentage of 46,XX cells. An overview of the total number of vitrified oocytes of 33 women with TS and the percentage of 46,XX cells in lymphocytes or buccal cells are presented. Mainly women with >20% 46,XX cells had cumulatively ≥20 vitrified oocytes. Remarkably, some patients without 46,XX cell line were able to vitrify ≥20 oocytes. Green dots represent women with a 46,XX cell line, blue dots represent women with structural aberrations, and orange dots represent women with 45,X/47,XXX karyotype. Percentages of 46,XX cell line in buccal cells are indicated with *.

A significant correlation was found between the total number of vitrified oocytes and AMH (r = 0.54 and *p* < 0.01) (Figure 2B), AFC (r = 0.49 and *p* < 0.01), FSH (r = −0.65 and *p* < 0.01), and percentage 46,XX cells (r = 0.49 and *p* < 0.01) (Figure 4). No correlation was found between the total number of vitrified oocytes and estradiol level (r = −0.29 and *p* = 0.26) or age (*p* = 0.62 and *p* = 0.07).

Four women did not succeed in having vitrified oocytes (#10, #26, #31, and #33), due to poor response in the first and/or second cycle. They all had a baseline FSH >10 E/L, and three women had a low AMH as well (in one woman, AMH was not reported).

### 3.3. Pregnancy Outcomes

Pregnancies occurred in 10/33 (30%) women ± three years after their last vitrification cycle (range 1–10 years). Five of these had a 45,X/46,XX karyotype, three had a structural aberration, and two had a 45,X/47,XXX karyotype (Table 1). Most women reported having no active desire for children yet because of their young age or the fact that they did not have a partner. Eight women got pregnant spontaneously and two women got pregnant after a new IVF treatment. A new IVF treatment was performed in these two women as their ovarian reserve was still sufficient. So far, no one has used their vitrified oocytes to become pregnant. 

Pregnancy outcomes of 9/10 women were available for analysis (#13, #16, #17, #19, #20, #25, #29, #31, and #33). The mean age of the women during the first pregnancy was 27 ± 4.9 years. Two women experienced an early miscarriage, two women who conceived spontaneously chose to terminate the pregnancy (because of unwanted pregnancy and reason unknown), and five women had a full-term pregnancy. Comorbidities during pregnancy occurred in 3/9 women (33%), including pregnancy induced hypertension, diabetes gravidarum, and thyroid disease. Three out of five women gave birth spontaneously, one woman had a primary cesarean section due to spondylodesis, and one woman had a secondary cesarean section due to fetal distress and transverse position of the baby. Postpartum complications occurred in 1/5 women (20%) who had a postpartum hemorrhage. In total, two boys and three girls were born with a birthweight between 2130 and 3355 g. One girl was diagnosed with TS after birth (#29) and one child was born with esophagus atresia, which required surgery.

A second pregnancy occurred in 5/10 women (50%) (#13, #19, #20, #29, and #32). One woman had an early miscarriage and four women delivered a child. One woman developed pregnancy induced hypertension and one woman developed incipient pre-eclampsia. Three women gave birth spontaneously and one woman had a primary cesarean section because of a previous cesarean section. No complications arose peri- and postpartum. The children showed no visible congenital abnormalities.

Two women had a third pregnancy (#13 and #32), of which one developed hypertension during pregnancy. One woman gave birth spontaneously and one woman had a secondary cesarean section due to fetal distress. No complications developed peri- and postpartum. Both children were in good condition and showed no visible congenital abnormalities.

## 4. Discussion

Vitrification of mature oocytes is currently the most applied technique to preserve fertility in women with TS [10,17]. However, data on the outcome of stimulation procedures for oocyte vitrification and follow-up in this group is still limited. 

In this article, we report the results of oocyte vitrification in the largest cohort of women with TS to date (*n* = 33). Oocyte vitrification was successfully performed in 88% of the women with a median of six vitrified oocytes in the first cycle. By offering multiple stimulation cycles, the total number of vitrified oocytes can be increased. Baseline AMH, AFC, FSH, and percentage of 46,XX cells had a significant correlation with the cumulative number of vitrified oocytes. Spontaneous pregnancies and pregnancies after IVF occurred after the vitrification process. So far, none of the women in this cohort have used their vitrified oocytes to become pregnant.

### 4.1. Interpretation of the Findings

Our results are largely consistent with previous studies that have reported on vitrification outcomes in women with TS [13,14,15]. The majority of ovarian stimulations and oocyte retrievals were performed safely, as complications only occurred after 3/69 punctures. Similar to other studies, the mean age of women during the first stimulation cycle was 21 years [13,14]. In contrast with the study conducted by Martel et al., the number of cancelations prior to oocyte retrieval due to poor response in our group was small. This could be explained by the fact that in the current study mainly women with a 45,X/46,XX karyotype are included. It is known that women with a mosaic karyotype have a better reproductive outcome compared to women with 45,X karyotype [4,15]. 

The median number of vitrified oocytes in the first stimulation cycle in women with TS is in line with other studies that reported a mean/median of 4–9 vitrified oocytes per cycle [13,14,15]. Compared to women who cryopreserve oocytes before the start of an oncological treatment (mean 9.5 ± 6.1 oocytes per cycle), the number of vitrified oocytes per cycle is generally lower in women with TS due to the accelerated loss of follicles at a young age [2,18]. The recommended number of mature oocytes for a pregnancy in women without TS is approximately 10–20 [18,19,20]. Considering the high aneuploidy rate in ovarian cells in women with TS, the higher risk of miscarriage and chromosomal abnormalities in genetic offspring, and the possibility that oocytes will not survive the thawing process, it is evident that women with TS require significantly more oocytes for a reasonable chance of an ongoing pregnancy [5,6,21].

The optimal number of oocytes required for an ongoing pregnancy in women with TS remains unknown as studies on this topic are lacking. Currently, only one case of a woman with TS who became pregnant after using cryopreserved oocytes is reported [16]. In total, she had 29 vitrified oocytes, of which 13 oocytes were successfully fertilized, resulting in three blastocysts.

In our study, 73% of the women opted to undergo two or more stimulation cycles in order to increase the total number of vitrified oocytes. Ultimately, 52% of the woman successfully vitrified 20 oocytes or more, which is significantly higher than what has been reported in other studies [13]. This is most likely to be because only a small number of women underwent multiple stimulations in other studies and women with a high percentage of 46,XX cells are described in the current study.

Clear predictive markers to determine which women with TS are able to obtain an adequate number of vitrified oocytes per stimulation cycle are scarce. In contrast to our study, Talaulikar et al. (*n* = 7) and Brouillet et al. (*n* = 14) did not find a correlation between AFC or baseline AMH and the number of vitrified oocytes per cycle, probably due to their small sample sizes [13,14]. In clinical practice, AMH and AFC are often used to assess the ovarian reserve in women [10]. When both AMH and AFC are low, a low oocyte yield after ovarian stimulation is expected [22,23]. However, the predictive value of this marker regarding the outcome of oocyte vitrification in women with TS has yet to be determined [24,25]. It is important to be aware that AMH levels can vary due to inter-test and inter-cycle variability [26,27]. Moreover, different assays for AMH were used by the hospitals. Therefore, our AMH levels should be interpreted with caution.

All women in whom oocytes could not be vitrified due to a low oocyte yield had an FSH >10 E/L, which is in line with other studies [13,15]. The FSH levels in our study should be interpreted with caution since FSH has not always been determined at the beginning of the menstrual cycle and different assays have been used. 

Overall, the percentage of 46,XX cells in lymphocytes/buccal cells had a significant positive correlation with the number of vitrified oocytes, but the interindividual variation was high. This is in line with the study undertaken by Brouillet et al. [13]. In our cohort, the reproductive outcomes of ovarian stimulations and pregnancies were very favorable due to the fact that most women had a high percentage of 46,XX cells present in peripheral cell lines. Although it is questionable how representative this cohort is within the overall group of women with TS, it is understandable that those who still have a sufficient ovarian reserve at a young age are able to preserve their fertility successfully.

In the systematic review of Brouillet et al., a combination of normal FSH, AMH > 1.13 ng/mL, and the presence of a 46,XX cell line was predictive of obtaining at least six cryopreserved oocytes in a single ovarian stimulation cycle [13]. This was also largely consistent with our data. The combination of parameters could be useful for clinicians during fertility counseling in women with TS. In addition, it is preferable to karyotype at least two peripheral cell lines to rule out whether a hidden mosaicism is present [28]. It is important to keep in mind that those without a 46,XX cell line could still be fertile enough to undergo ovarian stimulation successfully or become pregnant spontaneously as a wide variation is seen between karyotype in peripheral cells and ovarian cells [21,29]. Therefore, oocyte vitrification should also be considered in those without a 46,XX cell line and sufficient ovarian reserve.

The age at which oocyte vitrification should be offered to these girls/women is also up for debate. A few years ago, a case report was published describing that vitrification was successfully performed on a 7-year-old girl with TS [30]. Given the very young age, it is questionable whether a girl of that age can tolerate the physical and mental burden of such a treatment, especially if multiple stimulation cycles are required. Data on the psychological impact of oocyte vitrification in prepubertal girls is currently lacking.

None of the women with TS have used their vitrified oocytes yet to become pregnant. In other women who vitrified oocytes in the past (e.g., prior to cancer treatment or social freezing), only <10% returned to utilize their vitrified oocytes [19,20]. The main reason was that most women became pregnant spontaneously. In our study, a surprisingly high percentage of spontaneous pregnancies was observed compared to previous studies, which could be explained by the high percentage of 46,XX cells in our cohort. It is expected that those who have a sufficient ovarian reserve during ovarian stimulation are likely to become pregnant if pregnancy is pursued within a few years after ovarian stimulation. Interestingly, in our cohort pregnancies also occurred in women who did not have successfully vitrified oocytes. The high number of pregnancies could be a result of the fertility counseling that most women with TS received at an early age, making them aware that the window to conceive is small.

Cardiovascular comorbidities, diabetes gravidarum, and thyroid problems were reported problems during pregnancy in our cohort, which are common risks for women with TS [31]. Prior to fertility preservation, physicians must carefully consider whether it is safe for a woman with TS to become pregnant, especially when severe comorbidities, such as cardiovascular diseases, are present. Preconception consultation and adequate monitoring during pregnancy is mandatory to facilitate safe pregnancy in women with TS [9]. 

### 4.2. Considerations for Clinical Practice

Worldwide, girls and women with TS are not routinely informed about their impaired fertility and/or referred for fertility counseling, while this topic is one of their biggest concerns [8,31]. Girls with TS and their parents should be informed about this matter at an early age. A referral to a fertility specialist is essential to discuss the chance of spontaneous pregnancy, fertility preservation options (OTC and oocyte vitrification), and alternative options such as oocyte- and embryo donation, adoption, foster care, or remaining childless [7]. The safety of pregnancy in women with Turner syndrome remains a matter of concern. Girls and women with TS should be informed about the increased risk of complications during pregnancies, and that having vitrified oocytes or ovarian cortex tissue does not guarantee a future pregnancy [5,7]. Furthermore, the option of psychological support should be mentioned and offered when needed.

Oocyte vitrification should be discussed with girls/women with TS who have a sufficient ovarian reserve and no contra-indication for pregnancy as it remains uncertain when premature ovarian insufficiency will occur. Physicians should mention that multiple stimulation cycles are likely to be required to obtain a sufficient number of vitrified oocytes for a reasonable chance of a later pregnancy [5,6,13]. Furthermore, the option of preimplantation genetic testing should be discussed as well. By selecting embryos without aneuploidy, the risk of miscarriage can be reduced [32,33]. However, this procedure also requires a large number of oocytes and embryos for selection, making the feasibility of this option uncertain [32,33].

The appropriate timing for fertility preservation should be assessed with the girl and their parents. Information about fertility preservation should be adjusted to the level and age of the girl to engage them in the discussion. Furthermore, scheduling multiple appointments could be helpful to avoid hasty decision-making [34]. 

Oocyte vitrification should preferably be performed when the girl is mature enough to understand the procedure, can make adequate decisions, and spontaneous menarche has occurred. OTC should only be offered in a research setting and with caution to those with a sufficient ovarian reserve, preferably prepubertal girls, until more is known about the long-term consequences of OTC in girls with TS and live births have been reported [21,28]. The psychological wellbeing of young girls deserves special attention and responsibility from healthcare providers during counseling and fertility preservation treatment.

### 4.3. Strengths and Limitations

To the best of our knowledge, this study describes the largest cohort of women with TS who have had vitrified oocytes and gives an overview of the follow-up after vitrification. The generalizability of the results increased by combining data of multiple centers in the Netherlands.

The retrospective design is the most important limitation of this study. In some women, information about hormone levels prior to stimulation and time to pregnancy was missing. Furthermore, our results should be interpreted with caution as our cohort includes a selected population of women with mainly 45,X/46,XX karyotype and favorable reproductive outcomes.

## 5. Conclusions

In this article, we report the reproductive outcomes of women with TS undergoing oocyte vitrification, including follow-up. A median of six oocytes were vitrified after the first ovarian stimulation cycle. Baseline AMH, FSH, AFC, and the percentage of 46,XX cells showed a significant correlation with the cumulative number of vitrified oocytes. After vitrification, women became pregnant spontaneously or through IVF. So far, none of the women have used their vitrified oocytes to conceive. However, the follow-up period of our study remains too short to draw firm conclusions about the utilization of vitrified oocytes in women with TS.

Larger studies reporting the outcome of oocyte vitrification, including follow-up in women with TS, are needed to elucidate the optimal number of vitrified oocytes for a live birth.

## Figures and Tables

**Table 1:** Characteristics of women with Turner syndrome.

Patient No.	Karyotype Lymphocytes (L)/ Buccal Cells (B)	Age Counseling/ Stimulation (Years)	Comorbidity	Menarche	AFC (*n*)	FSH (E/L)/ AMH (µg/L) **	Stimulation Rounds (*n*)	Total FSH (IE)	Vitrified Oocytes (*n*)	Pregnancy (*n*)	Age First Pregnancy (Years)	Children (*n*)
1	L: 45,X (18%)/46,XX (82%)	16/18	Cardiac	Yes	40	NR/5.10	4	3845	31	-		-
2	L: 45,X (18%)/46,XX (82%)	16/17	-	Yes	48	5.10/4.00	1	2100	23	-		-
3	L: 45,X (56%)/46,XX (44%) B: 45,X/46,XX	17/18	Thyroid Psychological	Yes	12	5.30/6.70	3	5250	26	-		-
4	L: 45,X (9%)/46,XX (91%)	17/17	-	Yes	10	7.40/1.90	2	1650	19	-		-
5	L: 45,X (3%)/46,XX (97%)	17/18	Psychological	Yes	17	9.00/2.00	3	8925	20	-		-
6	L: 45,X (27%)/46,XX (73%) B: 45,X (57%)/46,XX (43%)	18/18	-	Yes	NR	2.40/0.35	2	3150	3	-		-
7	L: 45,X (66%)/46,XX (34%)	18/18	Pulmonal	Yes	13	3.10/2.80	3	5625	23	-		-
8	L: 45,X (17%)/46,XX (83%)	18/19	-	Yes	16	4.00/4.60	4	7050	27	-		-
9	L: 45,X (7%)/46,XX (93%) B: 45,X/46,XX	19/19	Renal	Yes	9	5.30/2.40	2	1800	29	-		-
10	L: 45,X (81%)/46,XX (5%)/47,XXX (14%)	19/20	Cardiac	Yes *	1	43.23/0.06	1	5625	0	-		-
11	L: 45,X (54%)/46,XX (46%) B: 45,X (65%)/46,XX (35%)	21/21	-	Yes	35	NR	3	3680	38	-		-
12	L: 45,X (17%)/47,XXX (83%) B: 45,X (15%)/46,XX (11%)/47,XXX (74%)	21/21	Gastrointestinal	No *	1	17.00/0.03	3	10,125	6	-		-
13	L: 45,X (66%)/46,XX (34%)	22/22	Cardiac	Yes	35	NR	2	2000	6	Natural conception (3)	NR	2
14	L: 45,X (67%)/47,XXX (33%) B: 45,X (11%)/46,XX (4%)/47,XXX (85%)	22/22	-	Yes	9	3.10/1.11	3	7875	20	-		-
15	B: 45,X (9%)/46,XX (91%)	25/25	-	Yes	13	NR/1.30	3	6500	28	-		-
16	L: 45,X (35%)/46,XX (65%)	25/26	Cardiac	Yes	5	NR	2	3600	8	IVF (1)	29	1
17	L: 45,X (14%)/46,XX (86%)	26/26	-	Yes	11	6.70/1.60	3	6525	21	Natural conception (1)	30	1
18	L: 45,X (13%)/46,XX (87%)	27/28	Psychological	Yes	20	NR/3.10	3	9450	29	-		-
19	L: 45,X (37%)/46,XX (63%)	29/30	Cardiac Thryoid	Yes	16	NR/8.40	3	14,700	21	IVF (1) Natural conception (1)	33	2
20	L: 45,X (50%)/46,XX (50%)	29/30	-	Yes	15	NR	1	2475	27	Natural conception (2)	24	1
21	L: X-deletion	17/17	-	Yes	3	6.90/0.50	3	9225	9	-		-
22	L: 45,X (43%), 46,X, r(X) (57%)	17/17	Musculoskeletal	Yes	12	NR/2.46	2	1100	26	-		-
23	L: 45,X (75%)/46,X, r(X) (25%)	17/19	-	Yes	0	15.00/1.79	1	3000	3	-		-
24	L: X-deletion	17/17	-	Yes *	5	NR/0.20	1	2700	6	Natural conception (1)	20	NR
25	L: X-deletion	17/18	Cardiac	Yes	18	NR/1.00	1	2100	5	Natural conception (1)	23	0
26	L: 45,X (87%)/46,X, r(X) (13%) B: 45,X/46,X, r(X)	17/18	-	Yes	26	11.10/0.90	2	2700	cancel	-		-
27	L: Ring chromosome	20/21	-	Yes	8	NR/1.06	2	2925	6	-		-
28	L: Isochromosome B: Isochromosome	25/25	-	Yes	14	6.40/NR	3	11,100	23	-		-
29	L: Isochromosome B: Isochromosome	30/30	-	Yes	11	6.10/NR	3	7200	24	Natural conception (2)	33	2
30	L: 45,X/47,XXX	15/16	Cardiac	Yes	3	2.60/0.14	1	2475	1	-		
31	L: 45,X/47,XXX B: 45,X/47,XXX	17/17	-	Yes	3	26.70/NR	1	5400	0	Natural conception (1)	27	1
32	L: 45,X (93%)/47,XXX (7%)	19/19	-	Yes	NR	5.90/1.60	3	6750	14	Natural conception (3)	22	1
33	L: 45,X (3%)/47,XXX (97%)	19/20	Cardiac Gastrointestinal	Yes *	NR	20.00/0.02	1	4725	cancel	-		-

* Note: These women with TS eventually needed hormone replacement therapy. ** Note: Hormone levels were determined during counselling and have not always been determined at the beginning of the menstrual cycle. NR = not reported, AFC = antral follicle count, FSH = follicle stimulating hormone, AMH = anti-Mullerian hormone, and IVF = in vitro fertilization.

**Table 2:** Serum levels of FSH, AMH, and estradiol per karyotype prior to oocyte vitrification.

Karyotype Hormone Levels *	45,X/46,XX (*n* = 20)	Structural Aberrations (*n* = 9)	45,X/47,XXX (*n* = 4)
*n* FSH * (E/L), median (IQR)	*12* 5.3 (3.3–8.6)	*5* 6.9 (6.3–13.0)	*4* 13.0 (3.4–25.0)
*n* AMH (µg/L), median (IQR)	*16* 2.2 (1.2–4.6)	*7* 1.0 (0.5–1.8)	*3* 0.1 (Not applicable)
*n* Estradiol (pmol/L), median (IQR)	*11* 190 (4–266)	*3* 96 (Not applicable)	*2* 260 (Not applicable)

* Note: Hormone levels were determined during counselling and have not always been determined at the beginning of the menstrual cycle. FSH = follicle stimulating hormone and AMH = anti-Mullerian hormone.

## Data Availability

Pseudonymized patient data can be made available upon reasonable request.
